# Cost of hospitalization for firearm injuries by firearm type, intent, and payer in the United States

**DOI:** 10.1186/s40621-017-0120-0

**Published:** 2017-07-19

**Authors:** Corinne Peek-Asa, Brandon Butcher, Joseph E. Cavanaugh

**Affiliations:** 1 The Department of Occupational and Environmental Health, College of Public Health, University of Iowa, 145 North Riverside Drive, S143 CPHB, Iowa City, IA 52242 USA; 2 The Department of Biostatistics, College of Public Health, University of Iowa, Iowa City, Iowa USA

**Keywords:** Firearm violence, Hospitalization costs, National sample

## Abstract

**Background:**

Firearm injuries disproportionately affect young, male, non-White populations, causing substantial individual and societal burden. Annual costs for hospitalized firearm injuries have not been widely described, as most previous cost studies have focused on lifetime costs. We examined a nationally-representative database of hospitalizations in the US to estimate per-hospital and overall hospital costs for firearm injuries by intent, type of weapon, and payer source.

**Methods:**

We conducted a retrospective cohort study of all firearm injury hospitalizations in the National Inpatient Sample from 2003 through 2013. The National Inpatient Sample, maintained by the Healthcare Utilization Project, is a stratified and weighted national sample of more than 20% of all hospitals. All admissions for firearm injuries were identified through Ecodes, yielding a weighted total of 336,785 for the study period. Average annual per-patient and overall hospital costs were estimated using generalized linear modelling, controlling for patient and hospital variables. Costs by intent, firearm type, and payer sources were estimated.

**Results:**

Annually from 2003 through 2013, 30,617 hospital admissions were for firearm injuries, for an annual rate of 10.1 admissions per 100,000 US population. More than 80% of hospitalizations were among individuals aged 15–44, and rates were nine times higher for males than females and nearly ten times higher for the Black than the White population. More than 60% of admissions were for assaults, and 70% of the injuries that had a known firearm type were from handguns. The average annual admission cost was $622 million. The highest per-admission costs were for injuries from assault weapons ($32,237 per admission) and for legal intervention ($33,462 per admission), but the highest total costs were for unspecific firearm type ($373 million) and assaults ($389 million). A quarter of firearm injury hospitalizations were among the uninsured, yielding average annual total costs of $155 million.

**Conclusion:**

Hospitals can project that government insurance will be the highest source for firearm injury reimbursement, and depending on healthcare access laws, that many of their firearm injury admissions will not be covered by insurance.

## Background

Among all injury deaths in the United States in 2015, 17% were caused by a firearm (National Center for Injury Prevention and Control & US Centers for Disease Control and Prevention, 2015; Karp, 2011). Among youth aged 15–24 this percent increased to 29.6%, and for Black youth firearms were the leading overall cause of death, resulting in 58.6% of all injury deaths. Individual firearm ownership is high but not unique to the United States, although the burden of firearm violence is: World Health Organization mortality data from 2010 indicate that US gun-related homicide rates were 25 times higher than in a comparison group of 23 high income countries (Cook & Ludwig, 1997; Council on Injury, Violence, and Poison Prevention Executive Committee & American Academy of Pediatrics, 2012; Grinshteyn & Hemenway, 2016). While the majority of research has been on firearm deaths, the individual, family, and community burden is also high for those with non-fatal firearm injuries.

Research to reduce the health and societal burden of firearm injuries has been lacking, and medical and public health groups have published calls to build an evidence base for prevention (Karp, 2011; Branas et al., 2017; Strong et al., 2016). Understanding the economic burden of firearm injuries is a critical element of advancing our knowledge base. Of seven studies reporting the cost burden of firearm injuries (Salemi et al., 2015; Spitzer et al., 2017; Corso et al., 2007; Lee et al., 2014; Miller, 2012; Fowler et al., 2015; Allareddy et al., 2012), two have estimated hospitalization costs for firearm injuries in the US population (Salemi et al., 2015; Spitzer et al., 2017). Salemi et al. (2015) estimated annual hospitalization costs at $679 million and reported hospitalization costs by patient and hospital (Salemi et al., 2015). Spitzer et al. (2017) estimated total hospitalization costs for the period of 2006–2014 at $6.6 billion and reported costs by payer source (Spitzer et al., 2017). Neither of these studies conducted multivariable analysis to examine cost components from firearm injuries. Of the other firearm cost studies, three focused on the overall lifetime costs associated with firearm deaths and injuries (Corso et al., 2007; Lee et al., 2014; Miller, 2012), one focused on lifetime medical costs and productivity loss (Fowler et al., 2015), and one that examined hospital costs included only children (Allareddy et al., 2012).

The aim of this study was to estimate annual and per-hospitalization cost for firearm injuries by intent, weapon type, and payer source, controlling for patient and hospital characteristics. We examined data from the National Inpatient Sample from 2003 through 2013. The healthcare industry will benefit from information about the annual direct medical costs associated with firearm injuries and the main contributors to these costs, as this information can be used to anticipate costs and to compare to a national benchmark. Information about patient characteristics, injury intent, type of weapon, and payer source will alsobe helpful, but these relationships have not been examined routinely in previous studies. These data will also be helpful to those planning intervention programs by bringing more attention to the economic burden of firearm injuries in the US.

## Methods

This retrospective cohort study included patients admitted to the hospital for a firearm injury, identified through the National Inpatient Sample from 2003 through 2013. The National Inpatient Sample (NIS) is maintained by the Health Care Utilization Project (HCUP) and is the largest all-payer inpatient care database in the United States (Agency for Healthcare Research and Quality, 2012). Prior to 2012, the NIS approximated a 20% stratified sample of hospitals; beginning in 2012, the NIS is comprised of a stratified sample of discharges from all hospitals participating in HCUP. Our analyses incorporated sampling weights appropriate for each year in the study period.

We identified firearm injuries through External Cause of Injury Codes (Ecode 922.0–985.4). The NIS database provides four variables populated with Ecodes, and a firearm code in any of these four variables was included in the sample. Approximately 15% of NIS patient files that have an injury diagnosis are missing an Ecode, and thus we investigated the potential for firearm injuries to be entered without an Ecode (Greenspan et al., 2006). We first identified cases with an ICD-9 Injury Diagnosis Code (ICD 800–999) that had all Ecode variables missing. We then compared the distribution of primary injury diagnoses among injury cases missing an Ecode with those identified as firearm injuries. The most common overlapping diagnosis codes were open abdominal wounds (1.1% of firearm injuries vs. 0.02% of non-Ecode injuries) and open brain injury with deep coma (1.1% of firearm injuries vs. 0.002% of non-Ecode injuries). Based on the lack of overlap in injury diagnosis distributions overall, and the very small proportion of both samples with overlapping diagnoses, we concluded that firearm injuries are infrequently missing Ecodes in the NIS data. Firearm injuries were characterized through Ecodes by intent (unintentional, self-harm, assault, legal intervention/terrorism, and undetermined) and firearm type (handgun, shotgun, hunting rifle, assault weapon, and other/unspecified).

National population-based rates were calculated by age, sex, and race, using US Census Data annual estimates as the rate denominator. Since race was missing on 17% of the retrospective sample, single imputation of race was done using a baseline-category logit regression using costs and patient and hospital level variables as described above as predictors. Since the NIS codes race and ethnicity differently than the Census, we re-categorized the Census classifications of race and ethnicity to match that of NIS. Specifically, the Census classifications of Not Hispanic White, Black, American Indian or Alaskan Native, Asian or Native Hawaiian Pacific Islander, and two or more races were categorized as White, Black, Native American, Asian or Pacific Islander, and other respectively to match the NIS race classifications. Lastly, the Census Hispanic category was classified to match the NIS Hispanic race category.

The main outcome was hospital costs, which better reflect the actual costs for the hospitalization than the hospital charges provided in the NIS dataset. Costs were obtained by a conversion of charges using the cost-to-charge ratios files provided by the NIS. These files contain a multiplier specific to each hospital per year that is applied to the patient-level charges. The NIS-provided user-guides were followed in converting charges to costs (https://www.hcup-us.ahrq.gov/db/state/costtocharge.jsp#how). Costs were adjusted for inflation to Q4 of 2013 for inpatient hospital services based on the Bureau of Labor Statistic’s Consumer Price Index.

We used multivariable modelling to estimate the association of patient-level costs by intent, firearm type and payer source, controlling for covariates and hospital characteristics. Covariates were chosen to minimize the model QIC score and included sex, age, race, income, hospital disposition (routine vs. other), died during hospitalization, payer, hospital bed size, hospital region, and year. Year was treated as a categorical variable because it did not have a linear trend. Variables such as hospital length of stay and severity were not included because they were intermediates and greatly increased the QIC score. A generalized linear model (GLM) based on the gamma distribution with a log link function was found to best fit the data. The model was fit using generalized estimating equations with an exchangeable working correlation matrix to account for data collected longitudinally at each hospital. In the NIS, hospitals are nested within each sampling stratum, yet the hospital composition of each stratum may change from year to year. NIS sampling weights were also incorporated into the GLM to obtain national estimates. All analyses were conducted using SAS, version 9.4. The univariable summary statistics were compiled using the SURVEYFREQ procedure and the multivariable modeling results were obtained using the GENMOD procedure. Cost estimates for intent, firearm type, and payer source were obtained from the multivariable model using the BYLEVEL option in conjunction with the LSMEANS statement. As such, the model-based cost estimates were adjusted for quantitative and qualitative covariates by using observed means and observed relative frequencies, respectively.

## Results

### Rate of firearm injury hospital admissions

A weighted total of 336,785 hospital admissions for firearm admissions were identified from 2003 through 2013 in the NIS sample, for an annual average number of 30,617 and a rate of 10.1 admissions per 100,000 US population (Table 1). More than 80% of hospitalizations were among individuals aged 15–44, with the highest annual rate of 28.9 per 100,000 among those aged 15–24 years. The rate of firearm injury for males (18.2 per 100,000) was nine times higher than for females (2.1 per 100,000). Nearly half of all hospitalizations were among the Black population, who experienced a rate of 39.7 per 100,000. The race category of “other” had the second highest rate of 21.1 per 100,000, and this group includes primarily people with multiple race and ethnicity designations.

**Table 1:** National estimates of the total number, percent, and average annual rate (per 100,000) of firearm injuries by age, sex, and race National Inpatient Sample, 2003–2013

Variable	Total (95% CI)	Percent (95% CI)	Average annual rate (per 100,000)
Overall	336,785 (299, 653–373,919)	−	10.1
Age			
< 5	1020 (799–1242)	0.3% (0.2, 0.4)	0.5
5–14	6685 (5762–7609)	2.0% (1.8, 2.2)	1.5
15–24	136,282 (119,859–152,705)	40.6% (39.6, 41.6)	28.9
25–44	141,089 (125,114–157,065)	42.0% (41.3, 42.7)	15.6
45–64	40,756 (36,597–44,915)	12.1% (11.6, 12.7)	4.8
65+	10,081 (9140–11,023)	3.0% (2.8, 3.2)	2.3
Sex			
Male	298,307 (264,860–331,755)	89.3% (88.9, 89.7)	18.2
Female	35,818 (32,175–39,462)	10.7% (10.3, 11.1)	2.1
Race/Ethnicity			
White	94,564 (85,790–103,339)	28.5% (26.3, 30.8)	4.4
Black	162,824 (139,762–185,885)	49.1% (46.3, 52.0)	39.7
Hispanic	56,917 (45,368–68,465)	17.2% (14.5, 19.8)	11.1
Asian or Pacific Islander	3356 (2783–3930)	1.0% (0.9, 1.2)	2.1
Native American	2110 (1422–2798)	0.6% (0.4, 0.8)	8.5
Other	11,626 (9626–13,626)	3.5% (3.0, 4.0)	21.1

**Fig. 1:**
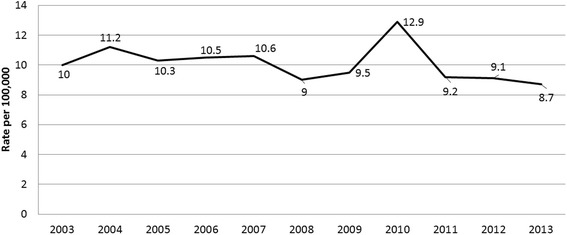
Age-adjusted national firearm hospital admission rates, US, National Inpatient Sample, 2003–2013

Annually, the trend in firearm injury rates did not differ substantially from 2003 to 2013. While slightly decreasing from 10.01 in 2003 to 8.7 in 2013, the rate fluctuated throughout the period, reaching a peak of 12.9 in 2010 (Fig. 1).

### Demographic characteristics and payer source by firearm injury intent

Overall, 60.6% of firearm injuries were assaults, 22.6% were unintentional, and 8.6% were self-harm (Table 2). Among children, firearm injuries of unintentional intent were the most common, comprising 55.8% of firearm injuries to children aged five and younger and 42.4% of those aged five to 14. From the ages of 15 through 64, assault was the most common intent, followed by unintentional injuries. The proportion of firearm injuries caused by self-harm increased with age, reaching 38.8% among those aged 65 and over. Among this age group, 32.8% of firearm injuries were unintentional. Although assault was the most common intent for both males and females, a higher proportion of males (61.5%) than females (52.8%) were due to assault.

**Table 2:** Association of Patient Characteristics with Hospital-Admitted Firearm Injuries by Intent, National Inpatient Sample, 2003–2013

	Intent of firearm injury				
	Unintentional	Assault	Self harm	Legal intervention	Undetermined
	N (row %)	N (row %)	N (row %)	N (row %)	N (row %)
Overall	76,203 (22.6)	204,104 (60.6)	29,027 (8.6)	6224 (1.8)	21,227 (6.3)
Age					
< 5	570 (55.8)	362 (35.4)	15 (1.4)	0 (0)	76 (7.4)
5–14	2837 (42.4)	3171 (47.4)	259 (3.8)	37 (0.5)	384 (5.7)
15–24	27,784 (20.4)	93,041 (68.3)	5228 (3.8)	1803 (1.3)	8426 (6.2)
25–44	30,126 (21.4)	87,606 (62.1)	10,788 (7.6)	3254 (2.3)	9315 (6.6)
45–64	11,387 (27.9)	17,229 (42.3)	8787 (21.6)	973 (2.4)	2381 (5.8)
65+	3304 (32.8)	2108 (20.9)	3913 (38.8)	149 (1.5)	608 (6.0)
Sex					
Male	67,113 (22.5)	183,338 (61.5)	23,240 (7.8)	5844 (2.0)	18,771 (6.3)
Female	8525 (23.8)	18,899 (52.8)	5718 (16.0)	346 (1.0)	2331 (6.5)
Race					
White	32,433 (34.3)	31,031 (32.8)	22,932 (24.3)	2697 (2.9)	5472 (5.8)
Black	28,830 (17.7)	118,048 (72.5)	2423 (1.5)	1907 (1.2)	11,616 (7.1)
Hispanic	10,121 (17.8)	40,931 (71.9)	1983 (3.5)	1187 (2.1)	2694 (4.7)
Asian or Pacific Islander	638 (19.0)	2282 (68.0)	223 (6.6)	619 (1.8)	153 (4.6)
Native American	666 (31.5)	1074 (50.9)	179 (8.5)	51 (2.4)	142 (6.7)
Other	2264 (19.5)	7568 (65.1)	837 (7.2)	195 (1.7)	763 (6.6)
Payer					
Medicare	5809 (31.6)	5216 (28.4)	5552 (30.2)	412 (2.2)	1392 (7.6)
Medicaid	17,853 (19.5)	62,287 (68.1)	4715 (5.2)	1128 (1.2)	5487 (6.0)
Private including HMO	21,326 (28.9)	37,344 (50.6)	9573 (13.0)	1175 (1.6)	4354 (5.9)
Self-pay	21,569 (21.3)	64,552 (63.7)	6274 (6.2)	1898 (1.9)	7108 (7.0)
Other	9646 (18.6)	34,704 (67.0)	2913 (5.6)	1611 (3.1)	2885 (5.6)

Chi-square tests for sex, race, & payer all have a *p* value <0.0001. Chi-square tests for age could not be computed since age < 5 for legal intervention/terrorism has 0 counts, but the text removing this age category had a *p* value <0.0001

The proportion of firearm injuries caused by assaults was far higher for non-Whites than Whites, and was the intent in more than 70% of all firearm injuries among Blacks and Hispanics. Whites had the highest proportion of unintentional (34.3%) and self-harm (24.3%) firearm injuries.

About half of firearm hospital admissions among those with private insurance were from assault, compared with 68.1% of those insured by Medicaid, 63.7% of those without insurance. Firearm admissions among those insured by Medicare had a very different distribution than other payer sources, having the highest percentage of unintentional (31.6%) and self-harm (30.2%) firearm injuries and the lowest percentage of assault injuries (28.4%).

### Demographic characteristics and payer source by type of weapon

For 59.4% of firearm hospital admissions the type of weapon was not known within the medical record source (Table 3). Most of the injuries with a known weapon type were caused by handguns, accounting for 31.2% of all injuries and 76.8% of injuries with a known weapon type. Handguns comprised a higher proportion of injuries among children five and under and elderly age 65 and over. A higher proportion of females (35.0%) than males (30.8%) were injured by handguns. Whites and those in the ‘other’ race/ethnicity category had the highest proportion of injuries from handguns. Medicare-covered firearm injuries had the lowest proportion of unknown weapon type (47.3%), and the highest proportion of injuries from shotguns (8.7%) and rifles (4.3%).

**Table 3:** Association of Patient Characteristics with Hospital-Admitted Firearm Injuries by Type of Firearm, National Inpatient Sample, 2003–2013

Type of weapon					
	Handgun	Shotgun	Rifle	Assault weapon	Other/Unspecified
	N (row %)	N (row %)	N (row %)	N (row %)	N (row %)
Overall	105,119 (31.2)	24,059 (7.1)	6726 (2.0)	914 (0.3)	199,968 (59.4)
Age					
< 5	420 (41.1)	77 (7.5)	24 (2.3)	0 (0)	502 (49.1)
5–14	1956 (29.3)	758 (11.3)	397 (5.9)	10 (0.1)	3565 (53.4)
15–24	39,255 (28.8)	8960 (6.6)	1696 (1.2)	308 (0.2)	86,063 (63.2)
25–44	43,703 (31.0)	9789 (6.9)	2654 (1.9)	458 (0.3)	84,485 (59.9)
45–64	15,023 (36.9)	3466 (8.5)	1451 (3.6)	88 (0.2)	20,727 (50.8)
65+	4527 (44.9)	949 (9.4)	495 (4.9)	45 (0.4)	4066 (40.4)
Sex					
Male	91,808 (30.8)	21,049 (7.1)	5968 (2.0)	787 (0.3)	178,695 (60.0)
Female	12,528 (35.0)	2811 (7.8)	745 (2.1)	122 (0.3)	19,614 (54.8)
Race					
White	37,251 (39.4)	9309 (9.8)	5599 (5.9)	384 (0.4)	42,021 (44.4)
Black	43,552 (26.7)	8427 (5.2)	347 (0.2)	341 (0.2)	110,158 (67.7)
Hispanic	16,955 (29.8)	4682 (8.2)	453 (0.8)	138 (0.2)	34,689 (60.9)
Asian or Pacific Islander	963 (28.7)	223 (6.6)	26 (0.8)	10 (0.3)	2135 (63.6)
Native American	578 (27.4)	228 (10.8)	90 (4.2)	10 (0.4)	1206 (57.2)
Other	4205 (36.2)	795 (6.8)	102 (0.9)	28 (0.2)	6498 (55.9)
Payer					
Medicare	1474 (39.4)	1596 (8.7)	791 (4.3)	59 (0.3)	8703 (47.3)
Medicaid	5459 (29.7)	6515 (7.1)	1096 (1.2)	187 (0.2)	56,544 (61.8)
Private including HMO	5164 (34.4)	5742 (7.8)	2760 (3.7)	187 (0.3)	39,698 (53.8)
Self-pay	6206 (30.1)	6994 (6.9)	1410 (1.4)	200 (0.2)	62,304 (61.4)
Other	3047 (28.7)	3212 (6.2)	671 (1.3)	281 (0.5)	32,720 (63.2)

Chi-square tests for sex, race, & payer all have a *p* value <0.0001. Chi-square tests for age could not be computed since age < 5 for legal intervention/terrorism has 0 counts, but the text removing this age category had a *p* value <0.0001

### Costs for firearm injury hospitalizations by intent, type of weapon, and payer source

The average hospital admission costs for firearm injuries exceeded $622 million per year, which represents a small portion of overall firearm death and injury costs. Results from the multivariable model estimating annual costs per patient and total annual costs, controlling for age, race, income, disposition, death during hospitalization, year, and hospital bed size and region are presented in Table 4.

**Table 4:** Estimated Annual Cost per Hospital Stay and Total Hospital Costs, by Firearm Type, Intent, and Payer Source, National Inpatient Sample, 2003 - 2013^a^

Variable	Level	Annual costs per admission (US$)	95% CI for annual costs per admission	Total annual costs (US$) (rounded to thousands)	95% CI for total annual costs (rounded to thousands)
^b^Firearm type	Handgun	19,175	18,495–19,880	183,241,000	176,743,000–189,979,000
	Hunting Rifle	20,770	18,918–22,804	12,699,000	11,567,000–13,944,000
	Assault Firearm	32,237	24,199–42,945	2,676,000	2,009,000–3565,000
	Other/Unspecified Firearm	20,530	19,753–21,339	373,213,000	359,088,000–387,920,000
	Shotgun	23,026	21,530–24,627	50,362,000	47,090,000–53,864,000
^c^Intent	Unintentional	16,975	16,038–17,967	117,595,000	111,104,000–124,467,000
	Assault	20,989	20,080–21,941	389,449,000	372,583,000–407,113,000
	Legal Intervention	33,462	30,018–37,301	18,933,000	16,985,000–21,106,000
	Self-Harm	24,369	22,918–25,913	64,305,000	60,476,000–68,380,000
	Undetermined	18,953	17,623–20,383	36,574,000	34,008,000–39,334,000
^d^Payer source	Medicaid	24,714	23,539–25,949	205,508,000	195,737,000–215,778,000
	Medicare	21,834	20,227–23,568	36,485,000	33,799,000–39,382,000
	Other	21,020	19,900–22,202	98,911,000	93,640,000–104,473,000
	Private/HMO	19,772	18,977–20,601	132,602,000	127,270,000–138,161,000
	Uninsured	16,816	16,107–17,556	155,014,000	148,479,000–161,836,000

^a^Age, sex, race, income, disposition, died,, payer, hospital bed-size, hospital region, & year (treated as categorical)

^b^All pairwise comparisons statistically significant except for Handgun vs. Hunting Rifle

^c^All pairwise comparisons statistically significant

^d^All pairwise comparisons statistically significant except for Medicare vs. Private/HMO

For admissions for which the type of firearm was known, handguns accounted for over 70% of the costs, which exceeded $183 million. This was the highest total admission cost per known firearm type, although handgun injuries were the least costly per admission. Assault weapons, although the most expensive at over $32,000 per admission, had the lowest annual costs at $2.7 million. Admissions from shotguns, which had the second highest cost per admission, had the second highest total annual cost of over $50 million. All pairwise comparisons by type of firearm were statistically significant with *p* < 0.05 with the exception of handgun vs. hunting rifle.

By intent, legal intervention had the highest per-admission average cost of $33,463, followed by self-harm at $24,369. Admissions from assaults had an average per-admission cost of $20,989 but accounted for $389 million in average annual costs – by far the highest overall cost by any intent. Admissions from unintentional firearm injuries were the second highest overall annual cost at over $117 million. All pairwise comparisons were statistically significant with *p* < 0.05.

Among payer sources, Medicaid had both the highest per-admission average at $24,714 and the highest total annual average of more than $205 million. Uninsured patients accounted for an annual average of $155 million. In sum, government and uninsured admissions from firearm injuries accounted for nearly $400 million in average costs per year, or 65% of all firearm admission costs. All pairwise comparisons by type of firearm were statistically significant with *p* < 0.05 with the Medicare vs. Private/HMO.

## Discussion

Average annual direct medical costs for hospital-admitted firearm injuries exceeded $622 million for the years of 2003 through 2013. This is an underestimate of the overall direct hospitalization costs because readmissions for further treatment may not have been identified with an Ecode noting the original cause of the injury. While this represents less than 1% of the overall costs of $377 billion for all hospital stays in the US, firearm injuries represent relatively high costs per patient (Moore et al., 2014). In 2012, the average cost per hospital stay was $10,400 (Moore et al., 2014), which is less than half of the average costs for a firearm injury. The lowest average cost for a firearm-related admission was $16,975 for an unintentional firearm injury, and the highest were $32,237 for an injury from an assault weapon and $33,462 from legal intervention.

In 2012, uninsured patients comprised 5% of all hospitalizations, while we found that from 2003 to 2013, an average of 30.1% of firearm injury hospitalizations were among the uninsured (Moore et al., 2014). In contrast, 29% of all hospitalizations were paid by private insurance compared with 21% of firearm hospitalizations. Based on their per-hospital cost, the overall hospitalization cost, and the low proportion of private insurance coverage, firearm injuries pose a high burden on our healthcare system.

Our cost estimates provide only a small component of the overall burden of firearm injuries. Studies reporting lifetime medical and productivity costs range from $48 to $175 billion (Corso et al., 2007; Lee et al., 2014; Miller, 2012; Fowler et al., 2015; Allareddy et al., 2012), with differences based on the source of firearm incidence data, types of firearm injuries included, sources of cost estimates, and types of costs included in the estimate. Lost productivity from premature death as well as work time loss was the largest component of these costs, with a smaller proportion from direct medical care.

Studies that focus on medical care also exhibit substantial variation in methods and estimated cost burden. Four studies report the lifetime medical care costs from firearm injuries that occur in 1 year, and these range from an average cost of $684 million for hospital admissions in 2000 (Fowler et al., 2015) to an average cost of $1.5 billion for hospital admissions in 2010 (Miller, 2012). These studies vary in their definition of medical care costs, but most cover all costs, including transport, readmission, and nursing care, and some estimate lifetime rather than annual costs (Corso et al., 2007; Lee et al., 2014; Miller, 2012; Fowler et al., 2015; Allareddy et al., 2012).

The two main sources for estimating non-fatal firearm injuries are the National Inpatient Sample, used here, and the Consumer Product Safety Commission’s National Electronic Injury Surveillance System – All Injury Program, which estimates national firearm injury hospitalization rates based on 66 hospital Emergency Departments (Schroeder & Ault, 2001; U.S. Consumer Product Safety Commission, 2000). Although these hospitals are chosen to represent a range of hospitals by size, the sample over-represents urban emergency departments. Estimates of firearm injuries from the National Inpatient Sample tend to be lower than those from the National Electronic Injury Surveillance System. For example, Lee et al., used the National Inpatient Sample to estimate an annual average of 28,249 hospital admissions from 2006 through 2010, and in comparison Fowler et al., estimated an average of 36,224 per year from 2010 through 2012. Our annual average of 30,617 was closer to other estimates from the National Inpatient Sample.

We found that firearm injuries disproportionately affect youth, males, and a non-White population. These trends have been widely reported in previous research (Fowler et al., 2015; Allareddy et al., 2012; Leventhal et al., 2014). In addition, we found that assaults caused more than 60% of admissions, followed by unintentional injuries. These trends are also similar to those reported in other studies of non-fatal firearm injuries (Fowler et al., 2015; Leventhal et al., 2014). However, because firearms are such a lethal means of suicide, suicide comprises a higher proportion of firearm mortality by intent (Fowler et al., 2015). Rates of firearm hospital admissions did not change significantly from 2003 through 2013. Previous studies have found that hospitalizations decreased between 1998 and 2011 among children injured by a firearm (Kalesan et al., 2016), and for all firearm hospitalizations from 2000 to 2010 (Kalesan et al., 2013).

Handguns and assaults posed the largest overall hospitalization cost burden. Although the highest per-hospital cost was for assault weapons, handguns comprised over 70% of firearm injuries among those for which the weapon type was known and the total annual hospitalization costs were nearly $183 million. Assaults had the third-highest cost per hospitalization and were the most frequent intent, costing an annual total of nearly $373 million. Two other studies reported costs by injury intent. Fowler et al. reported lifetime medical and lost work time costs among those admitted to a hospital to be approximately $475 million for unintentional firearm injuries, $600 million for self-harm/suicide, and over $2 billion for assault/homicide (Fowler et al., 2015). Corso et al. reported the lifetime costs medical costs for assaults as $800 million and for self-harm as $124 million (this study included only violent firearm injuries) (Corso et al., 2007). While these study’s estimates are higher due to factors described above, the trends by intent are similar.

No prior studies have reported costs associated with the type of firearm, although information about the type of firearm would be helpful to prioritize intervention approaches. One major challenge to estimating costs by firearm type is the high prevalence of hospitalizations for which the firearm type is unknown. In the National Inpatient Sample, nearly 60% of the firearm types were unknown. Handguns accounted for nearly 70% of all hospitalizations for which weapon type was known, and among these handguns accounted for four times the total annual hospitalization costs than for any other type of firearm.

In addition to a high prevalence of missing information about firearm type, this study has other limitations. Approximately 25% of injuries in the National Inpatient Sample are missing Ecodes, and it is possible that these could contain some firearm injuries. Our examination of injury diagnoses among admissions without Ecodes suggested that firearm injuries likely comprise less than 1% of those missing Ecodes. Our sample also does not include readmissions that did not have an Ecode identifying a firearm as the cause of injury, and thus our findings are likely an underestimate. The extent of missing data for the type of firearm limits our ability to fully examine injury and cost characteristics. The NIS is the largest and most representative sample of US hospitalizations, but may be subject to sampling bias and not designed specifically as a firearm injury surveillance system. Our analysis includes only hospitalized firearm injuries, which are about one third of all reported firearm injuries.

## Conclusion

These findings demonstrate the high healthcare cost burden of firearm injuries. Firearm disproportionately impacts a young, minority, and uninsured population. Hospitals can expect that the largest proportion of treatment for firearm injury admissions will be reimbursed by public sources, and, depending on trends in health insurance coverage, that a fifth of patients will have no insurance. While individual treatment costs will be highest for assault weapon injuries and legal intervention, overall costs will be highest for handguns and assaults. Efforts to prevent firearm injuries, particularly among assaults and injuries caused by handguns, could reduce this cost burden.

## Abbreviations

Ecode: External Cause of Injury Code
NIS: National Inpatient Sample
